# Supplementary material for: The Neurokinin-1 Receptor Is a Target in Pediatric Rhabdoid Tumors
Source: Curr Oncol. 2021 Dec 26;29(1):94–110. doi: 10.3390/curroncol29010008 (PMC8775224; doi:10.3390/curroncol29010008)
Supplement: Supplementary file 1 [file curroncol-29-00008-s001.zip › Kolorz et al. Supplementary Data Figure S1.pdf]

a)

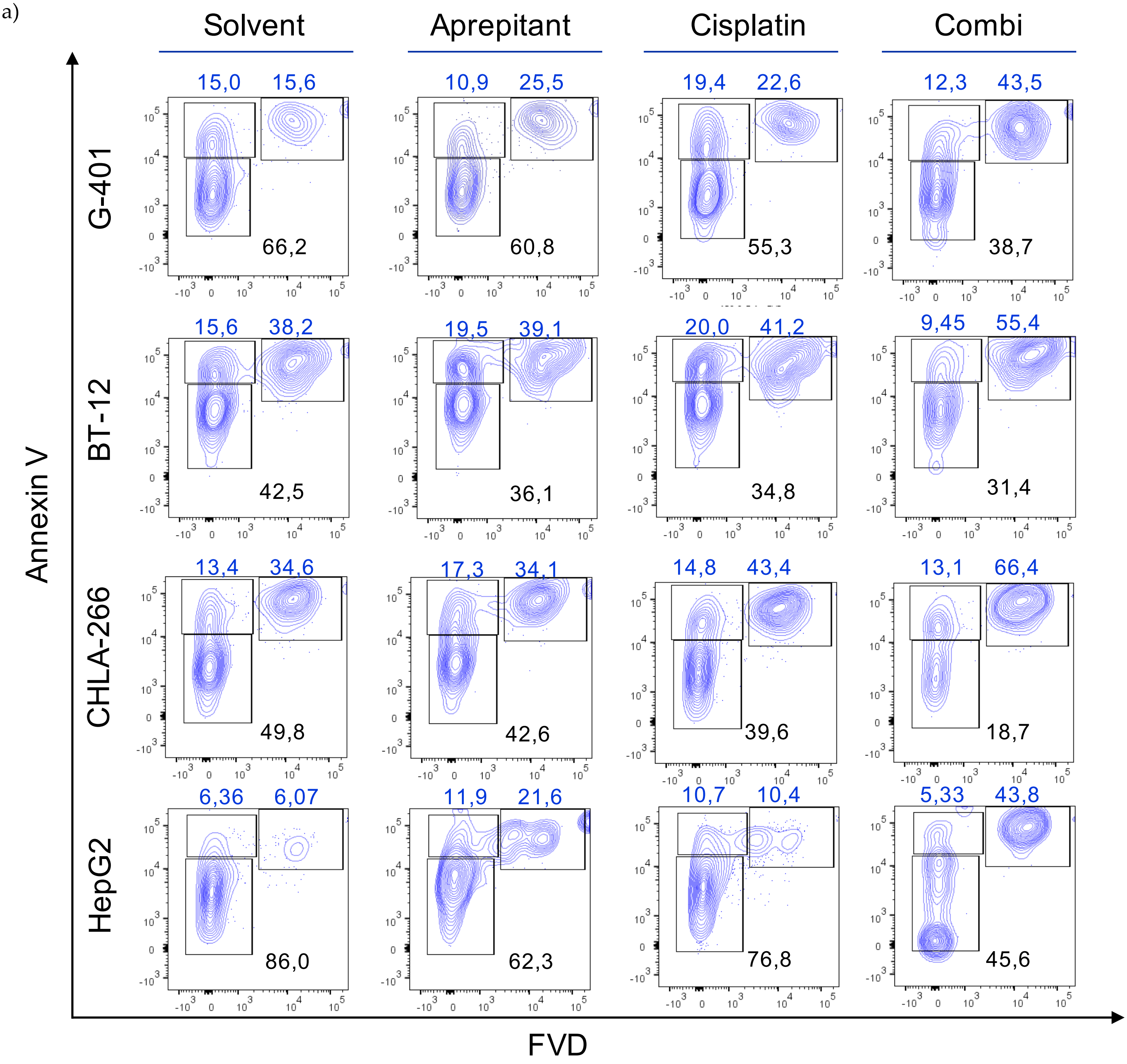

Supplementary Data Figure S1. (**a**) Determination of apoptotic cell populations of G-401, BT-12, CHLA-266 and HepG2 was performed by staining with annexin V and fixable viability dye (FVD) and assessed through fluorescence-activated cell sorting. Cells were treated with aprepitant (Apre, 15-50 μM), cisplatin (Cis, 20 μM) and aprepitant + cisplatin (Combi) for 48h. DMSO was used as treatment control. Shown is the second representative of three experiments. Numbers represents the percentages of the cell populations; black: viable; blue: apoptotic
