# Supplementary material for: The Neurokinin-1 Receptor Is a Target in Pediatric Rhabdoid Tumors
Source: Curr Oncol. 2021 Dec 26;29(1):94–110. doi: 10.3390/curroncol29010008 (PMC8775224; doi:10.3390/curroncol29010008)
Supplement: Supplementary file 1 [file curroncol-29-00008-s001.zip › Kolorz et al. Supplementary Data Figure S3.pdf]

a)

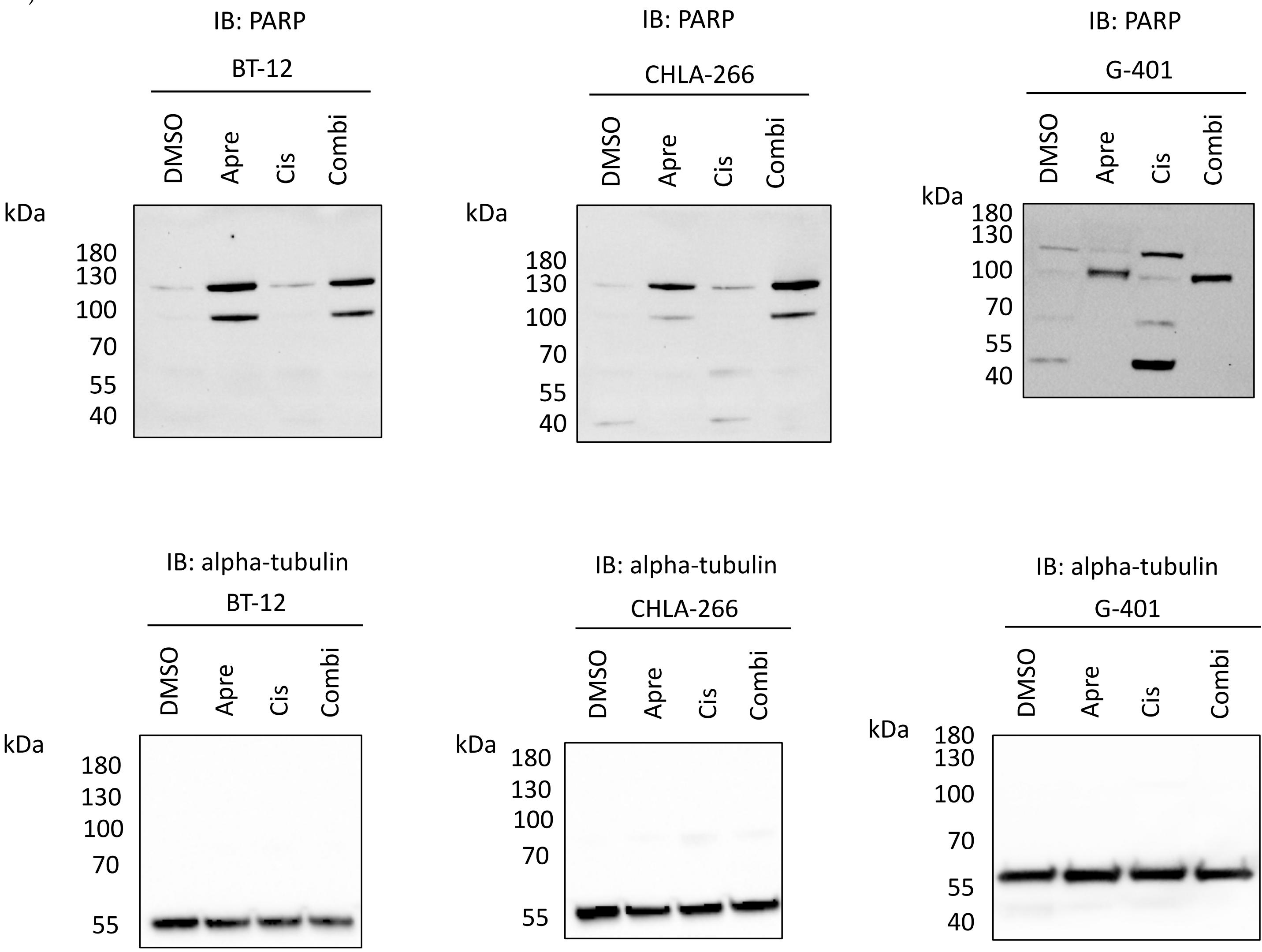

b)

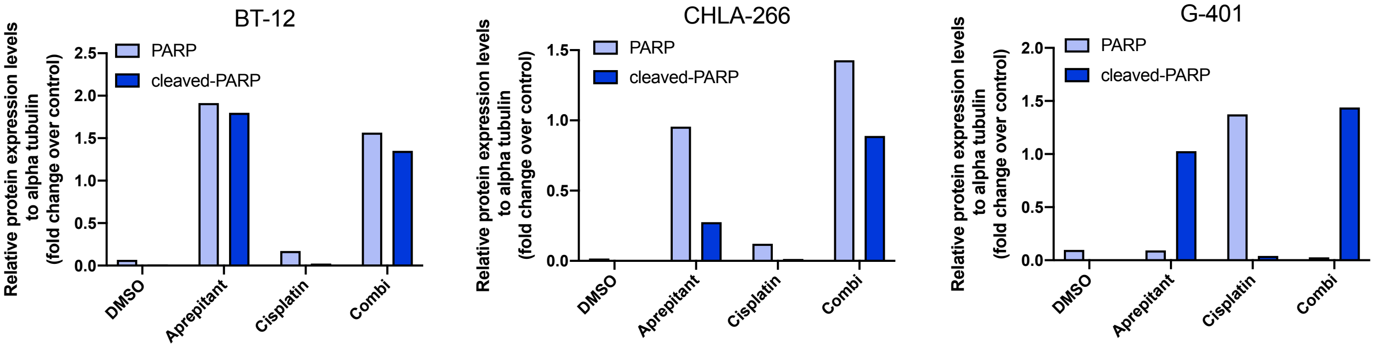

Supplementary Data Figure S3. (**a**)Western blot analysis of G-401, BT-12 and CHLA-266 upon treatment with aprepitant (APR, 40 -50 µM), cisplatin (CIS 20 µM) and aprepitant + cisplatin (COMBI) (48h). DMSO used as a treatment control. Shown is the first representative. Blots are representative of n=2. (**b**) Densitometry analysis of western blots. Relative protein expression levels were normalized to alpha tubulin expression.
